# Disentangling the contributions of agentic, antagonistic, and neurotic narcissism to drive for thinness and drive for muscularity

**DOI:** 10.1371/journal.pone.0253187

**Published:** 2021-06-15

**Authors:** Leonie Hater, Johanna Schulte, Katharina Geukes, Ulrike Buhlmann, Mitja D. Back

**Affiliations:** Institute of Psychology, University of Münster, Münster, Germany; University of Padova, ITALY

## Abstract

Body image concerns revolving around body ideals (thin ideal, muscular ideal) are widespread among women. Whereas a stronger preoccupation with ideal physical appearance is often assumed for narcissistic women, previous empirical findings have been mixed. Following a tripartite structure of *agentic*, *antagonistic*, *and neurotic* narcissism facets, we reexamined whether trait narcissism predicted *drive for thinness* and *drive for muscularity*. We further explored the role of importance of appearance as a mediator and moderator of the relation between narcissism and body image concerns. Latent structural equation modeling was applied to self-report data from two independent nonclinical female samples (*N*_Sample1_ = 224, *N*_Sample2_ = 342). Results underlined the importance of distinguishing between narcissism facets: Neurotic (but not agentic or antagonistic) narcissism uniquely predicted drive for thinness and drive for muscularity. Importance of appearance mediated but did not robustly moderate these relations. Hence, neurotic narcissistic women (characterized by hypersensitivity, shame, and a fragile self-esteem) are particularly prone to body image concerns. This vulnerability seems partly driven by how much importance they ascribe to their appearance. Future work might build on these insights to further unravel the processes linking neurotic narcissism to body image concerns and how these can be targeted in practical interventions.

## Introduction

Body image concerns emerge around societally established body ideals and reflect excessive cognitive and emotional preoccupation with one’s own shape and weight. They are alarmingly frequent among women [1–4]. But who is especially vulnerable to these concerns? Previous research has investigated narcissism as one potential predictor but has yielded inconsistent results (e.g., [5, 6]). The present work readdresses the question of whether narcissism predicts body image concerns and extends previous work by (a) including both drive for thinness and drive for muscularity as key body image concerns, (b) incorporating insights regarding the tripartite structure of narcissism, including agentic, antagonistic, and neurotic aspects, (c) exploring the potential moderating and/or mediating roles of importance of appearance, and (d) applying latent modeling (i.e., structural equation modeling; SEM) across two independent samples to gain more robust insights.

### Body image concerns: Drive for thinness and drive for muscularity

The most well-established female body ideal in most cultures and societies is the thin ideal [7–10]. It promotes a slim body shape [11] and low body weight [7, 12], but does not represent the average female body [7, 13]. Body image concerns that pivot on the thin ideal are labeled *drive for thinness* [7, 14]. Drive for thinness is related to negative consequences, including the engagement in dysfunctional-shape and weight-regulative behaviors [15, 16] and clinically significant eating disorder symptomatology [14, 17, 18].

The toned and muscular body ideal has also gained relevance among women [19–23]. It promotes extremely defined and visible muscles such as a “six-pack”, which, similar to the thin ideal, hardly reflects the average female body. Body image concerns about muscularity, labeled *drive for muscularity* [24], are related to excessive weight training and restrictive eating [8, 24, 25]. This can lead to impairments, to the development of eating disorders [26] and the development of muscle dysmorphia, a subtype of body dysmorphic disorder [27]. Drive for thinness and drive for muscularity can occur concomitantly [15, 22].

Proponents of the sociocultural perspective [28, 29], which includes the Tripartite Influence Model of body image disturbances [13], have argued that body image concerns develop from constant exposure to the body ideal from the media. Accordingly, drive for thinness results from the constant transmission of the thin ideal via social and mass media, conveying the idea that a thin body (for a woman) is socially desired and worth striving for [30]. Empirical support for the idea that exposure to the thin ideal through the media increases women’s drive for thinness came from two meta-analyses [31, 32] and a cross-cultural longitudinal study [33] that inspected effects of traditional media as well as a recent study that examined effects of social media [34]. The same seems to apply to the muscular ideal: In recent years, remarkably muscular female bodies have increasingly been depicted in the media [35, 36] and an experimental study showed that exposure to the muscular body ideal has led to increased muscularity-related body image concerns among women [20].

Despite the evidence that exposure to medial thinness- and muscularity-oriented ideals increases body image concerns, the sociocultural perspective falls short of explaining why not all women who are exposed to such media develop the respective concerns [37]. It is most likely that other, more individual factors contribute as well [38]. Specifically, the cognitive-behavioral model of body image postulates that certain personality traits reinforce (the development of) body image concerns [39]. For example, across several studies, neuroticism was identified as a risk factor for general body image concerns (e.g., [40, 41]) and perfectionism as a risk factor for thinness concerns (e.g., [42]). Here, we aim to shed light on the personality trait of narcissism as a potential predictor of both drive for thinness and drive for muscularity.

### Agentic, antagonistic, and neurotic facets of narcissism

The last couple of decades of research have produced different conceptualizations of narcissism with varying facets that differ in their intra- and interpersonal expression and functionality [43–45]. For a long time, the broadest consensus has centered around the distinction between *vulnerable* and *grandiose narcissism* [44, 46]. However, theoretical and empirical research has shown that these two broad factors entangle agentic and antagonistic content (grandiose narcissism) and antagonistic and neurotic content (vulnerable narcissism; [45, 47–52]). Consequently, contemporary narcissism research [45, 47, 49–52] tends to follow an alternative trifaceted structure, which distinguishes between (1) *agentic narcissism* (assertiveness, self-promotion, striving for social attention and admiration), (2) *antagonistic narcissism* (arrogance, devaluation and exploitation of others, striving for supremacy), and (c) *neurotic narcissism* (insecurity, shame, hypersensitivity to and avoidance of criticism). These three facets of narcissism are related to different self-regulatory dynamics and show distinct associations with a wide variety of intra- and interpersonal outcomes (for an overview, see [45]), but they have not yet been investigated in relation to body image concerns.

### The relations between narcissism and body image concerns

Why should narcissistic women be particularly concerned about their body image (e.g., their thinness and/or their muscularity)? Considering the facets of narcissism, the clearest expectations can be derived for neurotic narcissism: Being insecure, sensitive to criticism, and prone to experiencing shame should increase a person’s likelihood of developing body image concerns. Women with higher levels of neurotic narcissism need social approval to elevate and stabilize their rather low and fragile self-esteem [45, 53, 54]. Such approval could potentially be earned with an “ideal” body because achieving body ideals promises social rewards [55, 56]. Further, vulnerable narcissists’ hypersensitivity to criticism [57] likely increases body image concerns because a nonideal body potentially leads to (anticipated, assumed, and/or actual) devaluation by others [58, 59]. Finally, when exposed to body ideals, neurotic narcissists probably engage in self-protective strategies less often than agentic and antagonistic narcissists, for example, self-enhancement [47] or other-derogation [47, 53, 60]. This is crucial because these strategies may help narcissists to distance themselves from body ideals and the concomitant ego-threat potential. Neurotic narcissists might thus be more affected by ego threats from exposure to body ideals and may consequently be more prone to developing body image concerns. Taken together, from a theoretical perspective, needing validation from others, fearing their criticism, and lacking self-protective strategies render neurotic (more than agentic or antagonistic) narcissists vulnerable to body image concerns.

Empirically, there is evidence that vulnerable (but not grandiose) narcissism in women is positively related to body shame [61] and body dissatisfaction [62], whereas grandiose narcissistic women reported particularly positive body images [63]. Some studies have examined thinness-related concerns in particular [5, 6, 41, 64, 65] and the results have again varied as a function of the narcissism facet that was considered: Aspects allocable to vulnerable narcissism were positively related to drive for thinness; grandiose aspects had no or even protective effects on the degree of thinness concerns (for an overview, see also [66]).

So far, only a few studies have investigated the association of muscularity-oriented body image concerns and narcissism. One study found a more pronounced drive for muscularity in men with higher levels of grandiose narcissism [67], whereas another did not find such a correlation [55]. For women, grandiose narcissism was also significantly related to drive for muscularity, whereas vulnerable narcissism, on the other hand, was not [5]. To our knowledge, no previous study has considered the trifaceted structure of narcissism in the contexts of either drive for thinness or drive for muscularity. Moreover, the abovementioned studies (e.g., [5]) ran manifest regression analyses. Latent regression analyses (i.e., latent SEM), which remove measurement errors and provide less biased regression estimates, and, thus, allow for more robust results [68], have yet to be conducted.

### The role of importance of appearance

The assumption that neurotic narcissism goes along with increased levels of body image concerns seems to be grounded in the idea that body ideals (irrespective of whether they are matched by an individual’s appearance) have ego-threatening potential. This makes individuals strive for and worry about body ideals; particularly those individuals who are shame-prone, sensitive to social criticism, and in need of social approval, that is, those high in neurotic narcissism. This idea seems, however, only reasonable if an individual’s self-worth strongly depends on appearance-related aspects. However, the importance of appearance differs significantly between individuals [69] as individuals’ determination and evaluations of their self-worth are based on different domains. According to the contingency self-worth theory [70], physical appearance is only one of these domains of self-worth.

Cash et al. [69] demonstrated that women scoring higher on importance of appearance had a more negative body image. Similarly, Overstreet and Quinn [71] showed that basing self-worth on appearance-related approval from others was associated with increased dysfunctional preoccupation with one’s own body. Further, thinness [2] and muscularity concerns [29] have been associated with importance of appearance. At the same time, the higher individuals score on vulnerable narcissism, the more their self-worth appears to be contingent on their physical appearance [54]. Hence, importance of appearance plays a role in body image concerns (i.e., drive for thinness, drive for muscularity) on the one hand and in (vulnerable/neurotic) narcissism on the other.

These findings hint that importance of appearance might *mediate* or *moderate* the associations between neurotic narcissism and body image concerns. A mediating effect would imply that the feared ego-threat or aspired ego-boost effect of body ideal (mis-)match occurs in neurotic narcissistic women *because* they consider their appearance to be important. A moderating effect would imply that this effect occurs *the more* neurotic narcissistic women consider their appearance to be important. One previous cross-sectional study suggested that importance of appearance functions as a mediator between vulnerable narcissism and drive for thinness in women [5]. To our knowledge, no previous studies have conducted the respective mediation analyses with muscularity-related concerns and agentic, antagonistic, and neurotic narcissism or latent mediation analyses. We also did not find any previous studies that investigated whether importance of appearance moderates the effects of narcissism facets on drive for thinness or drive for muscularity. Mediator and moderator effects hence both require (further) exploration.

### The present research

Is narcissism related to body image concerns in women? The present work was aimed at providing new insights with respect to this question. We hereby built upon the latest insights into the three-faceted structure of narcissism involving agentic, antagonistic, and neurotic narcissism. Moreover, we expanded the focus of the analysis from body image concerns about thinness to concerns about muscularity, which have only recently gained relevance for women. Finally, we differentiated our analyses so that we considered importance of appearance as both a mediator and a moderator of the associations between narcissism and body image concerns.

By applying a latent analysis approach (i.e., latent SEM) instead of the more common manifest analyses and by analyzing data from two independent nonclinical female samples (Sample 1 Spanish speaking, Sample 2 German speaking), we aimed to provide comparatively robust results. We tested the hypothesis that neurotic narcissism would be found to be positively related to drive for thinness and explored the associations of drive for thinness with antagonistic and agentic narcissism. When we found significant associations between the narcissism facets and body image concerns, we further explored whether this was moderated and/or mediated by importance of appearance. Additionally, with Sample 2, we expanded the analyses to include drive for muscularity. That is, we investigated whether agentic, antagonistic, and neurotic narcissism were related to drive for muscularity and whether importance of appearance moderated and/or mediated the potential associations.

## Method

### Participants

Sample 1 consisted of *N* = 224 Spanish-speaking female participants recruited at the Complutense University of Madrid and via social network sites (e.g., Facebook). Participants’ ages ranged from 17 to 63 years with a mean of 32.26 (*SD* = 10.18). Half of them were university students (51%). Table 1 presents descriptive information regarding Body Mass Index (BMI) and eating disorder symptoms (i.e., Eating Attitudes Test scores; [72]).

**Table 1:** Body mass index, eating pathology, and body dysmorphic pathology.

	Sample 1		Sample 2		
	BMI	EAT 13	BMI	EAT 13	DCQ
*M*	24.63	7.32	22.78	7.63	6.57
*SD*	5.41	6.77	4.45	8.62	4.45
*Mdn*	23.53	5	21.80	5	6
Range	17.58–53.69	0–31	13.27–48.33	0–36	0–20
Clinical cut-off exceeded	0%	21%	3%	26%	7%

*Note*. BMI = Body Mass Index (kg/m^2^), EAT 13 = Eating Attitudes Test 13 items version, DCQ = Dysmorphic Concern Questionnaire. Cut-offs for clinically significant psychopathology: BMI < 17 (as one central criterion for Anorexia Nervosa, [27]), EAT 13 sum score > 10 (screening for disturbed eating behaviors; [72]), DCQ sum score > 14 (screening for body dysmorphic disorder; [73]).

For Sample 2, a total of *N* = 342 German-speaking female participants from 15 to 68 years of age (*M* = 28.18, *SD* = 10.92), recruited at the University of Münster and via social network sites, agreed to the use of their data and completed the online survey. Approximately two thirds (63%) were university students. Table 1 presents descriptive statistics for BMI, eating disorder symptoms, and body dysmorphic symptoms (i.e., Dysmorphic Concern Questionnaire scores; [74]). Sample 2 showed a somewhat lower average BMI than Sample 1, *t*(416.41) = 4.25 *p* < .001, but both samples did not differ in their mean level of disturbed eating behaviors, *t* (545.77) = -0.48, *p* = 0.633. Both samples show a typical non-clinical to sub-clinical distribution with very few participants exceeding clinical cut-offs for an anorectic BMI or body dysmorphic disorder, but around a fifth to a fourth of the samples exceeding the cut-off for disturbed eating behavior.

### Procedure

For both samples, data were collected using the online survey tool EFS Survey software [75]. At the beginning of the surveys, participants were informed about the topic and procedure of the study. Participants were asked for informed consent prior to completing the questionnaires. Participants in Sample 2 received curricular course credit for participation and, if they wished, brief feedback on their personality (Big Five) at the end of the survey. They could also take part in a raffle of 18 x 25€ online shop vouchers. On average, completing the surveys took 30 min in Sample 1 and 40 min in Sample 2. The study was approved by the Institutional Review Board at the University of Münster.

### Measures

The surveys consisted of self-report measures of personality and body image concerns, presented in Spanish (Sample 1) and German (Sample 2). Descriptive statistics (i.e., means, standard deviations, ranges, and internal consistencies) for each measure are reported in S1 Table. Both surveys contained further questionnaires, but only those relevant for this study are outlined below.

#### Body image concerns

Drive for thinness was measured with the identically labeled subscale from the Eating Disorder Inventory-2 (EDI DT; [76]). We used the Spanish [77] and German versions [78]. The seven items (e.g., “I am extremely afraid of gaining weight”) were answered on a 6-point scale ranging from 1 (*never*) to 6 (*always*).

Drive for muscularity (assessed only in Sample 2) was measured with the German version [79] of the Drive for Muscularity Scale (DMS; [24]). Because we were interested in concerns (but not behaviors), we used only the cognitive/emotional scale from the DMS. These seven items (e.g., “I wish I were more muscular”) were answered on a 6-point scale labeled the EDI DT.

#### Narcissism

To allow for a broad coverage of narcissism, we included a set of different narcissism measures. In Sample 1, we included three narcissism instruments: two broader instruments for the assessment of Grandiose Narcissism (the Narcissistic Personality Inventory (NPI [80] and the Narcissistic Admiration and Rivalry Questionnaire (NARQ; [47]), which both cover agentic and antagonistic aspects) and one for the assessment of Vulnerable Narcissism (the Hypersensitive Narcissism Scale (HSNS; [57]) that covers neurotic aspects). In Sample 2, we wanted to further increase the comprehensiveness of our assessment strategy and additionally included all four subscales of the short form of the Five-Factor-Narcissism-Inventory (FFNI; [81]) for the assessment of Vulnerable Narcissism (Reactive Anger and Distrust, which both cover antagonistic aspects, as well as Shame and Need for Admiration, which both cover neurotic aspects) to balance grandiose and vulnerable shares of narcissism. In the following, we describe how we have used these measures to capture agentic, antagonistic, and neurotic narcissism.

Agentic narcissism was assessed with the two agentic subscales (Leadership/Authority, LA; Grandiose Exhibitionism, GE) from the NPI [80]. We used the Spanish [82] and German versions [83]. The subscales contain 11 (NPI LA) and 10 (NPI GE) forced-choice items, for example, “I have a natural talent for influencing people” (NPI LA) and “I really like to be the center of attention” (NPI GE). They can be answered with a narcissistic (1) or a non-narcissistic answer (0). Agentic narcissism was additionally measured with the Admiration subscale (ADM) from the NARQ; [47] in Sample 2 and a Spanish translation of this questionnaire in Sample 1. The translation process is described in S1 Text. The Admiration subscale consists of nine items (e.g., “I show others how special I am”) answered on a 6-point scale ranging from 1 (*do not agree at all*) to 6 (*agree absolutely*).

Antagonistic narcissism was assessed with the antagonistic subscale (Entitlement/Exploitativeness, EE) from the NPI [80]. This subscale comprises four forced-choice items (e.g., “I insist upon getting the respect that is due me”). Antagonistic narcissism was further measured with the nine items (e.g., “I enjoy it when another person is inferior to me”) from the Rivalry subscale (RIV) of the NARQ [47]. In Sample 2, antagonistic narcissism was additionally assessed with two four-item subscales from a German version [84] of the FFNI [: Reactive Anger (RA; e.g., “I have at times gone into a rage when not treated right”) and Distrust (D; e.g., “I often think that others aren’t telling me the whole truth”). Answers were given on a 5-point scale ranging from 1 (*strongly disagree*) to 5 (*strongly agree*).

Neurotic narcissism was assessed with the Spanish [85] and German versions [86] of the HSNS. The HSNS consists of 10 items (e.g., “I often interpret the remarks of others in a personal way”) answered on a 5-point scale ranging from 1 (*not at all correct*) to 5 (*absolutely correct*). In Sample 2, neurotic narcissism was additionally assessed with two subscales from the FFNI [81]: Need for admiration (NFA; e.g., “I often feel as if I need compliments from others in order to be sure of myself”) and Shame (S; e.g., “When I realize I have failed at something, I feel humiliated”).

#### Importance of appearance

Importance of appearance was measured with the Spanish [87] and German versions [88] of the revised Appearance Schema Inventory (ASI; [69]). This questionnaire assesses self-evaluative (e.g., “How I look is an important part of who I am”) and motivational salience (e.g., “I try to be as physically attractive as possible”) with 20 items answered on a 5-point scale ranging from 1 (*disagree strongly*) to 5 (*agree strongly*).

### Analytical strategy

All analyses were conducted with the R Statistics program [89] by applying the *lavaan* package [90] for latent analyses. After inspecting the manifest intercorrelations of all subscales, in order to identify the unique predictors, we built several structural equation models as follows: We regressed the latent drive for thinness on latent agentic, antagonistic, and neurotic narcissism. This model was applied to Samples 1 and 2. We built a second model (applied only to Sample 2) in which the latent drive for muscularity was regressed on the three facets of narcissism. Indicators of the latent drive for thinness were the EDI DT items; indicators of the latent drive for muscularity were the DMS items. For latent agentic narcissism, we created mean variables by aggregating across the NPI LA, NPI GE, and NARQ ADM subscale items, respectively, and used them as indicators. Accordingly, for latent antagonistic narcissism, the means of the NPI EE, NARQ RIV, FFNI RA, and FFNI D subscales served as indicators. To create indicators for latent neurotic narcissism, we created mean variables by aggregating across the HSNS, FFNI NFA, and FFNI S items, respectively. We evaluated the model fits by computing four fit indices: the Comparative Fit Index (CFI), Tucker-Lewis Index (TLI), Root Mean Square Error of Approximation (RMSEA), and Standardized Root Mean Square Residual (SRMR). Sufficient model fit was defined as robust CFI > .95, robust TLI > .90, robust RMSEA < .08, and SRMR < .10 [91].

For narcissism facets that significantly predicted body image concerns, we extended the models by including a mediating effect of importance of appearance. Indicator variables for latent importance of appearance were the ASI items. We used bootstrapping to estimate confidence intervals for the mediation effects.

To explore the moderating effect of importance of appearance, we computed manifest interaction analyses for the narcissism facets that significantly predicted body image concerns in the SEM analyses. That is, we regressed drive for thinness on the main effects of and interaction between neurotic narcissism and importance of appearance for Samples 1 and 2, and we regressed drive for muscularity on the main effects of and interaction between neurotic narcissism and importance of appearance for Sample 2. We then inspected simple slopes for high (+ 1 *SD*), average (*M*), and low (-1 *SD*) levels of importance of appearance. To obtain manifest drive for thinness and drive for muscularity variables, we created mean variables across the EDI DT and DMS items. To obtain the manifest neurotic narcissism variable, we aggregated the *z*-standardized subscale mean variables (HSNS, FFNI NFA, FFNI S) into a single variable. For the importance of appearance variable, we aggregated across the items from the ASI-R. We did not conduct these analyses with latent variables due to the ongoing debate about reliability and the best practice for modeling latent interactions [92–94]. For latent and manifest analyses, we calculated standardized coefficients and set the significance level to *p* < .05. The complete data and R code can be obtained at https://t1p.de/osfnbic.

## Results

### Narcissism and drive for thinness

Table 2 presents the zero-order correlations between the narcissism facets and drive for thinness. Agentic narcissism was not related (Sample 1) or was negatively related (Sample 2) to drive for thinness. Antagonistic and neurotic narcissism had significant positive correlations with drive for thinness in both samples.

**Table 2:** Intercorrelations of (aggregated) measures applied to Sample 1 (upper triangle) and Sample 2 (lower triangle).

		1	2	3	4	5	6	7	8	9	10	11	12	13	14	15	16
**1**	**Agentic narcissism**		.80**	.78**	.86**	.35**	.30**	.32**	-	-	0.01	0.01	-	-	0.1	-	.27**
2	NPI LA	.78**		.37**	.58**	.21**	.20**	.17*	-	-	-0.08	-0.08	-	-	0.05	-	0.04
3	NPI GE	.78**	.36**		.52**	.29**	.23**	.28**	-	-	-	0.07	-	-	0.12	-	.36**
4	NARQ ADM	.84**	.51**	.51**		.35**	.29**	.33**	-	-	0.04	0.04	-	-	0.08	-	.25**
**5**	**Antagonistic narcissism**	.15**	.14**	.02	.19**		.88**	.88**			.63**	.63**			.27**	-	.43**
6	NPI EE	.19**	.19**	.09	.18**	.74**		.54**	-	-	.52**	.52**	-	-	.24**	-	.36**
7	NARQ RIV	.25**	.15**	.18**	.27**	.78**	.51**		-	-	.58**	.58**	-	-	.23**	-	.38**
8	FFNI RA	.19**	.12*	.08	.25**	.65**	.32**	.36**		-	-	-	-	-	-	-	-
9	FFNI D	-.22**	-.06	-.29**	-.17**	.58**	.20**	.28**	.11*		-	-	-	-	-	-	-
**10**	**Neurotic narcissism**	-.35**	-.28**	-.32**	-.24**	.50**	.25**	.41**	.26**	.45**		*a*	-	-	.43**	-	.54**
11	HSNS	-.23**	-.16**	-.23**	-.16**	.47**	.24**	.39**	.23**	.42**	.84**		-	-	.43**	-	.54**
12	FFNI NFA	-.39**	-.35**	-.30**	-.29**	.38**	.19**	.32**	.18**	.37**	.90**	.63**		-	-	-	.60**
13	FFNI S	-.29**	-.22**	-.30**	-.17**	.46**	.22**	.38**	.27**	.39**	.88**	.57**	.71**			-	-
**14**	**Drive for thinness**	-.17**	-.09	-.19**	-.12*	.28**	.16**	.25**	.13*	.23**	.47**	.32**	.47**	.44**		-	.60**
**15**	**Drive for muscularity**	.10	.10	.02	.13*	.28**	.20**	.26**	.18**	.13*	.26**	.24**	.21**	.21**	.28**		-
**16**	**Importance of appearance**	.02	-.06	.06	.05	.37**	.25**	.34**	.22**	.21**	.54**	.35**	.55**	.50**	.58**	.22**	

*Note*. Agentic, antagonistic, and neurotic narcissism reflect mean variables aggregated across each of their measures. NPI LA = Narcissistic Personality Inventory Leadership/Authority, NPI GE = Grandiose Exhibitionism, NPI EE = Entitlement/Exploitativeness, NARQ ADM = Narcissistic Admiration and Rivalry Questionnaire Admiration, NARQ RIV = Rivalry, HSNS = Hypersensitive Narcissism Scale, FFNI D = Five Factor Narcissism Inventory Distrust, FFNI NFA = Need for Admiration, FFNI RA = Reactive Anger, FFNI S = Shame. *a*: Would be 1 because in Sample 1, latent neurotic narcissism had only one indicator variable (HSNS).

*p < .05.

**p < .01.

**Fig 1:**
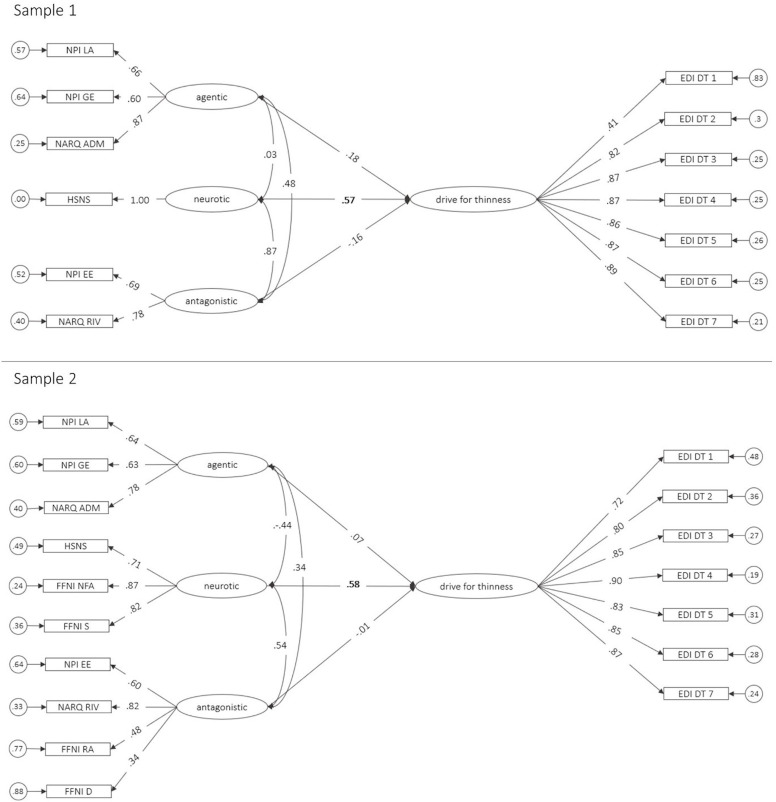
SEM of the relation between drive for thinness and the narcissism facets. NPI LA = Narcissistic Personality Inventory Leadership/Authority, NPI GE = Grandiose Exhibitionism, NPI EE = Entitlement/Exploitativeness, NARQ ADM = Narcissistic Admiration and Rivalry Questionnaire Admiration, NARQ RIV = Rivalry, HSNS = Hypersensitive Narcissism Scale, FFNI D = Five Factor Narcissism Inventory Distrust, FFNI NFA = Need for Admiration, FFNI RA = Reactive Anger, FFNI S = Shame, EDI DT = Eating Disorder Inventory Drive for thinness. Circles represent residual variances, rectangles represent manifest variables, ovals represent latent variables. Significant regression coefficients (*p* < .05) are printed in bold.

Structural equation models with drive for thinness as the latent dependent variable and the narcissism facets as the latent predictors had a good fit in Sample 1 (CFI = .983, TLI = .978, RMSEA [CI] = .046 [.022-.066], SRMR = .040) and a satisfactory fit in Sample 2 (CFI = .939, TLI = .926, RMSEA [CI] = .072 [.062-.082], SRMR = .070). In Sample 1, neurotic narcissism significantly predicted drive for thinness (β = .57, *p* = .006), whereas agentic (β = .16, *p* = .199) and antagonistic narcissism (β = -.16, *p* = .508) had no significant incremental effects (see Fig 1, upper panel). The total amount of variance explained by this model was 22%. In Sample 2, again, neurotic narcissism significantly predicted drive for thinness (β = .58, *p* = .001), whereas agentic (β = .07, *p* = .653) and antagonistic narcissism (β = -.01, *p* = .951) had no (incremental) effects (see Fig 1, lower panel). The total amount of variance explained by this model was 30%.

### Narcissism and drive for muscularity

**Fig 2:**
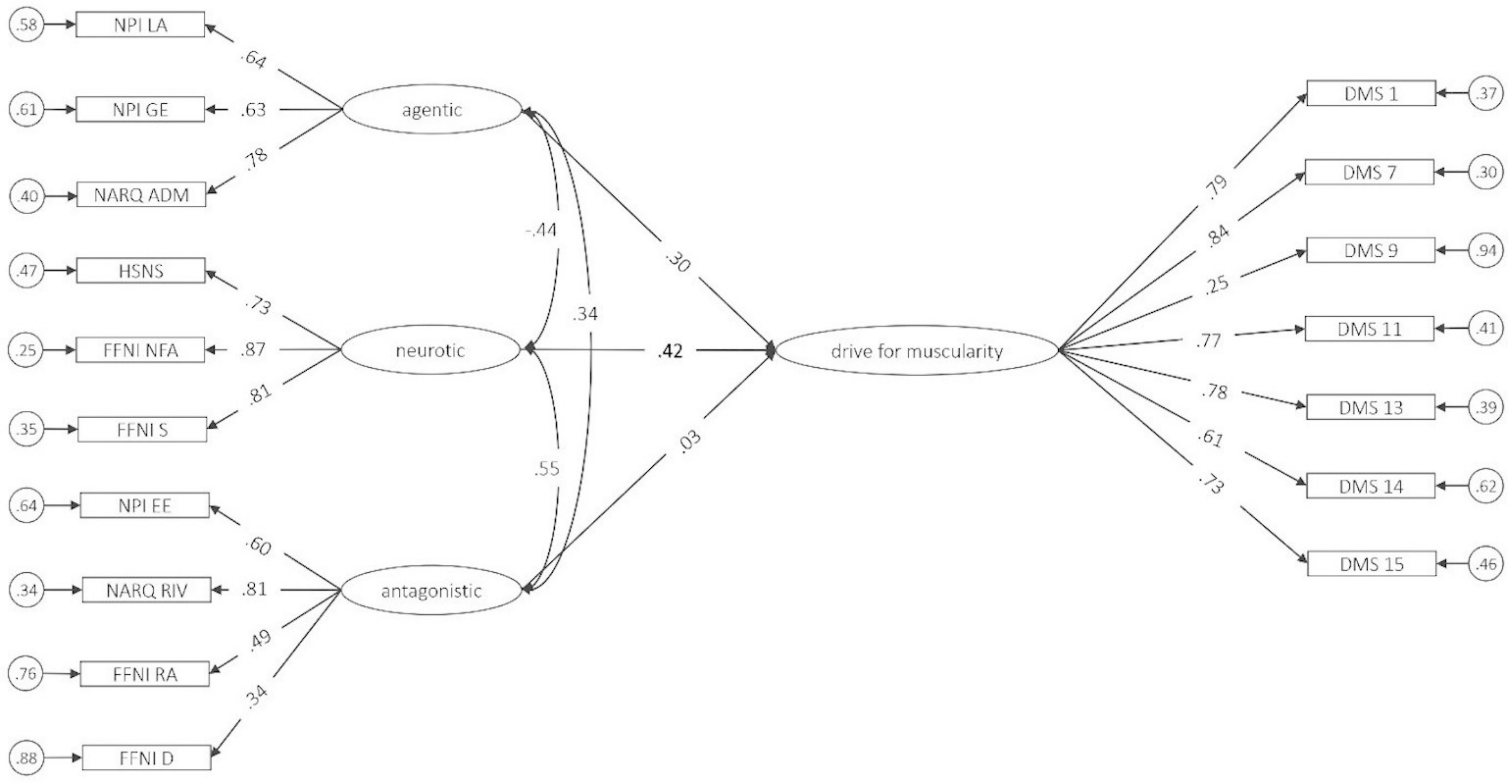
SEM of the relation between drive for muscularity and facets of narcissism in Sample 2. NPI LA = Narcissistic Personality Inventory Leadership/Authority, NPI GE = Grandiose Exhibitionism, NPI EE = Entitlement/Exploitativeness, NARQ ADM = Narcissistic Admiration and Rivalry Questionnaire Admiration, NARQ RIV = Rivalry, HSNS = Hypersensitive Narcissism Scale, FFNI D = Five Factor Narcissism Inventory Distrust, FFNI NFA = Need for Admiration, FFNI RA = Reactive Anger, FFNI S = Shame, DMS = Drive for Muscularity Scale. Circles represent residual variances, rectangles represent manifest variables, ovals represent latent variables. Regression coefficients (*p* < .05) are printed in bold.

Zero-order correlations indicated that agentic narcissism was not significantly related to drive for muscularity, whereas antagonistic and neurotic narcissism were. The structural equation model predicting drive for muscularity by the narcissism facets had a sufficient fit (CFI = .921, TLI = .905, RMSEA [CI] = .068 [.058-.078], SRMR = .068). As depicted in Fig 2, only the neurotic facet significantly and incrementally predicted drive for muscularity (β = .42, *p* = .025), whereas neither antagonistic narcissism (β = .03, *p* = .881) nor agentic narcissism (β = .30, *p* = .071) had incremental effects. A total of 17% of the variance in drive for muscularity was explained.

Supplemental analyses showed that controlling for BMI did not affect the pattern of results. Also, BMI did not moderate the effects of neurotic narcissism on drive for thinness or drive for muscularity (see S2 Table and S1 Fig). Also, results did not depend on whether individuals who exceeded EAT13 and BMI cut-offs for ED were included or excluded (see S2 Text).

### Mediating and moderating effects of importance of appearance

**Fig 3:**
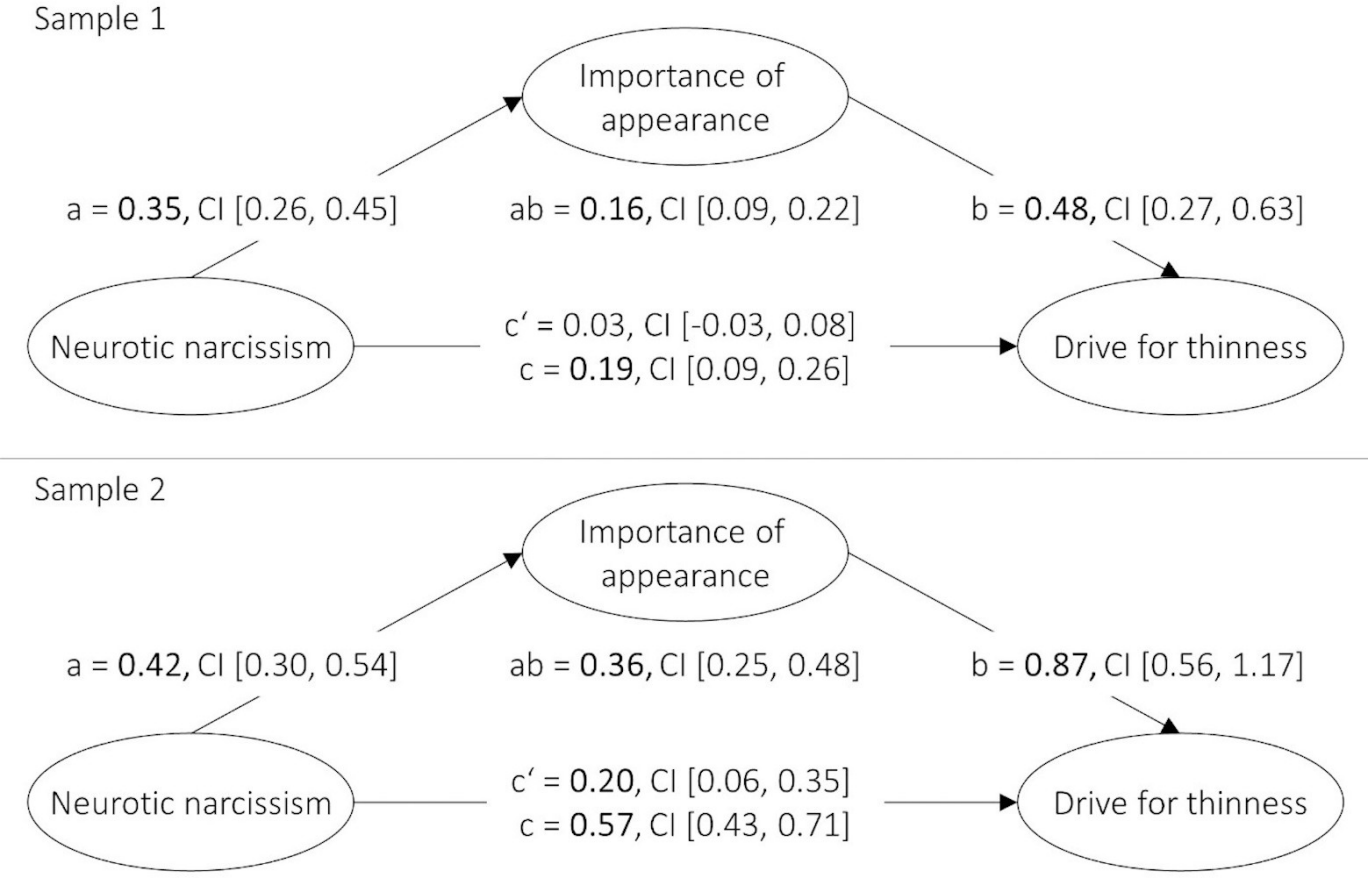
Mediating effect of importance of appearance on the relation between neurotic narcissism and drive for thinness. Neurotic narcissism, importance of appearance, and drive for thinness were latent variables (for indicator variables, see the Analytical Strategy). ab = indirect effect, c = direct effect, c’ = total effect. Confidence intervals (CI) were computed using bootstrapping. Path coefficients are unstandardized and printed in bold when significant (*p* < .05).

As outlined in Table 2, body image concerns, neurotic narcissism, and importance of appearance were positively associated with each other on a manifest correlational level. The results of the latent mediator analyses showed that importance of appearance mediated the relation between neurotic narcissism and drive for thinness in both samples (see Fig 3). By contrast, in a respective mediation model examining drive for muscularity, the path between importance of appearance and drive for muscularity was not significant (see S2 Fig).

Manifest moderator analyses yielded different results for Samples 1 and 2 (see Table 3): Whereas in both samples, neurotic narcissism and importance of appearance had significant main effects on drive for thinness, the interaction between these predictors was significant only in Sample 2. Thus, there was no robust evidence for an additional moderating effect of importance of appearance. Simple slope tests and illustrations are displayed in S3 Fig. Results for drive for muscularity (examined in Sample 2, see Table 3) indicated a significant main effect of neurotic narcissism, whereas the main and interaction effects of importance of appearance were not significant (for simple slopes, see S4 Fig). The total amount of variance explained by neurotic narcissism, importance of appearance, and their interaction was higher for drive for thinness (32% in Sample 1, 39% in Sample 2) than for drive for muscularity (8%).

**Table 3:** Manifest main and interaction effects of neurotic narcissism and importance of appearance on drive for thinness (Sample 1 and 2) and drive for muscularity (Sample 2).

	Sample 1		Sample 2			
	Drive for thinness		Drive for thinness		Drive for muscularity	
	β	*p*	β	*p*	β	*p*
Neurotic narcissism	**.16**	.015	**.23**	< .001	**.19**	.002
Importance of appearance	**.52**	< .001	**.46**	< .001	.12	.056
Neurotic narcissism x Importance of appearance	-.03	.616	**.11**	.005	-.07	.164
*R*² (adjusted)	38% (37%)		39% (38%)		8% (7%)	

## Discussion

The aim of our study was to investigate the relations between the personality trait narcissism and body image concerns in women. Specifically, we examined whether higher levels of agentic, antagonistic, and neurotic narcissism, respectively, were associated with an elevated drive for thinness and/or drive for muscularity. We therefore applied SEM to data from two (Spanish and German) nonclinical samples. Results showed that neurotic narcissism significantly predicted drive for thinness, but agentic and antagonistic narcissism did not. The same pattern arose for drive for muscularity. Importance of appearance mediated the association between neurotic narcissism and drive for thinness, that is, neurotic narcissists had more thinness-related concerns partially due to their higher valuation of appearance. Importance of appearance did not mediate the relation between neurotic narcissism and drive for muscularity, nor did it consistently act as a moderator (i.e., neurotic narcissism was not necessarily more strongly related to body image concerns for those who valued appearance more).

### Narcissism and drive for thinness

Previous studies have already investigated relations between narcissism and thinness-related body image concerns but did not account for the trifaceted structure of narcissism. In the current study, zero-order correlations showed that neurotic and antagonistic narcissism were significantly positively related to drive for thinness. These results support previous studies that suggested that vulnerable narcissism (reflecting the neurotic and antagonistic aspects) would be predictive of thinness concerns [5, 41]. Importantly, and consistent with our hypothesis, SEM analyses indicated that these associations were primarily driven by neurotic narcissism, which emerged as the strongest incremental predictor. Applying the tripartite structure of narcissism thus provided a way to differentiate across previous findings on the relation between narcissism and drive for thinness.

Why are neurotic narcissistic women particularly concerned about their thinness? The present work provides a first explanation by inspecting the role of importance of appearance: In line with past research [5], it lends support for the idea that neurotic narcissistic women consider their physical appearance to be particularly important and are therefore particularly concerned about their thinness. Future research might further explore this process jointly with at least three additional mechanisms: Neurotic narcissists’ (a) hypersensitivity, (b) low and fragile self-esteem, and (c) lack of ego-protective strategies.

Neurotic narcissistic hypersensitivity reflects a fear of others’ negative feedback and attempts to avoid disapproval [57]. Due to this sensitivity to criticism, neurotic narcissistic women might be more responsive to potential devaluation if they do not have an ideally thin body and thus more prone to respective dysfunctional concerns. Hence, increased drive for thinness in narcissistic women might be a consequence of their bias toward, fear of, and need to reduce criticism. On the contrary, agentic narcissists’ perception and attention processes are much less directed toward negative feedback; they “automatically inhibit threatening information” ([45], p. 6) and orient themselves more toward cues that support and correspond to their grandiose self-view (see also [95, 96]).

The level and stability of self-esteem in neurotic narcissists might also explain the association between neurotic narcissism and drive for thinness. Because neurotic narcissists’ self-esteem is typically low and fragile (e.g., [49]), they more strongly depend on others’ admiration and approval to stabilize it (e.g., [54]). Because striving for thinness is socially rewarded (e.g., [56]), neurotic (but not agentic or antagonistic) narcissistic women might hence use drive for thinness to stabilize their self-esteem.

Another potential explanation might be that, in contrast to agentic and antagonistic narcissists, neurotic narcissists engage less in the ego-protective strategies that can reduce experienced threat from exposure to and/or mismatching with the thin ideal. More precisely, agentic narcissists are strong in self-enhancement (e.g., self-promotion, favorable causal attribution; [47, 97, 98]). Hence, when confronted with a discrepancy between their own and “ideal” thinness, agentic narcissists would presumably focus on other, more “ideal” attributes (e.g., desirable personality; [96]) or reframe the mismatch in a self-enhancing manner. Antagonistic narcissists tend to react with other-derogating behaviors [45, 49]. Accordingly, in the presence of thinness-related ego threats, they might instead devaluate others (e.g., who were about to criticize them, who do not match the ideal either, or who convey the thin ideal). As opposed to engaging in these “active” ego-protective strategies of agentic and antagonistic narcissists, respectively, neurotic narcissists tend to instead react “passively” in the face of ego threats, for example, with shame or withdrawal [99, 100].

Taken together, neurotic (but not agentic or antagonistic) narcissism predicted drive for thinness in women, and this was partly due to finding that neurotic narcissistic women considered their physical appearance to be particularly relevant (to their self-worth). The typical (a) hypersensitivity, (b) low, fragile, and other-dependent self-esteem, and (c) lack of ego-protecting strategies provide further reasonable explanations that should be examined in future research.

We also explored a moderating effect of importance of appearance on the relation between neurotic narcissism and drive for thinness such that the relation was stronger the more importance women ascribed to their appearance. We found this in Sample 2 but not in Sample 1. More precisely, in Sample 1, an average level of importance of appearance seemed to increase the association, whereas a high level seemed to decrease the association, leading to a nonsignificant interaction effect. Thus, it remains inconclusive whether and how importance of appearance affects the strength of the association between neurotic narcissism and drive for thinness in women.

### Narcissism and drive for muscularity

Investigating not only drive for thinness but also drive for muscularity was of particular interest because muscularity-related body image concerns are a relatively new phenomenon among women. On a correlational level, drive for muscularity was significantly related to neurotic and antagonistic narcissism but not to agentic narcissism. Subsequent latent regression analyses identified neurotic narcissism as the strongest incremental predictor. This pattern is in contrast to Gordon and Dombeck’s previous study [5], which, using manifest regression analyses, suggested that grandiose narcissism (measured as a composite of agentic and antagonistic narcissism) predicts drive for muscularity, but vulnerable narcissism (measured similarly to neurotic narcissism) does not. However, the effects of vulnerable narcissism they found in that study were nearly significant and trended in the same direction as our results. It is thus presumable that neurotic narcissism plays a crucial role in determining drive for muscularity. In contrast to the previous findings by Gordon and Dombeck [5], we did not find a significant association between agentic narcissism and drive for muscularity. However, the respective slope in this study pointed in the same direction. Thus, it appears worthwhile for future studies to reinvestigate whether agentic narcissism is associated with drive for muscularity.

Our findings for drive for muscularity are consistent with the pattern found for drive for thinness. Accordingly, most of the previously outlined assumptions for why neurotic (but not agentic or antagonistic narcissism) predicts drive for thinness might be valid for drive for muscularity as well. That is, narcissistic women might be more preoccupied with matching the muscular body ideal due to (a) their sensitivity to and fear of others’ negative feedback, (b) their fragile self-esteem that depends on others’ admiration, and (c) their lack of alternative ego-maintaining strategies. However, the role of importance of appearance, which the present findings suggest partially explains the relation between neurotic narcissism and drive for thinness, seems different for drive for muscularity: Mediator analyses did not yield a significant path between importance of appearance and drive for muscularity. Therefore, it does not appear that increased muscularity-related concerns in neurotic narcissistic women can be explained by importance of appearance, but perhaps the increase can be explained by other motives or self-worth contingencies that have yet to be studied (e.g., perfectionism; [101]).

Similarly, the moderator analysis results were also different when investigating muscularity-related body image concerns: In the moderated regression model with neurotic narcissism, importance of appearance did not have a significant moderating effect or main effect on drive for muscularity. Hence, in spite of its significant association on a zero-order correlational level, which has similarly been reported in preliminary analyses [29], the role of importance of appearance seems rather secondary compared with neurotic narcissism in drive for muscularity as well as compared with its role in thinness concerns.

### Practical implications

The present work highlights the need to differentiate between the facets of narcissism to understand its relationship with body image concerns. In particular, neurotic narcissism was identified as a predictor of both drive for thinness and drive for muscularity. Moreover, the former association was (partially) mediated by importance of appearance. Thus, women who are narcissistically insecure and hypersensitive to social criticism might be at risk of body dissatisfaction, excessive appearance preoccupation, and body image pathology such as eating or body dysmorphic disorders, partially due to the tendency to base their self-worth on their appearance. To understand the development and maintenance of body image pathology, individual neurotic narcissistic tendencies should be considered. Likewise, these mechanisms could be targeted in psychotherapy, for example, by modifying appearance-related interpretation biases or supporting adaptive coping when faced with perceived criticism or ego threat. A positive body image should be fostered by distancing oneself from (medially transmitted) body ideals and by reducing appearance comparisons. Further, especially regarding drive for thinness, self-worth should be stabilized by domains other than appearance.

### Limitations and future research

Future research might build on the present work and address methodological and theoretical limitations. To begin with, we gathered cross-sectional data only. Thus, the results do not necessarily imply that neurotic narcissism *precedes* body image concerns. This is even more relevant for the mediator analyses, which suggested that neurotic narcissism fosters importance of appearance and therefore drive for thinness. Although our cross-sectional data were consistent with such causality assumptions, explicit tests of such assumptions require longitudinal or experimental data. The present findings paved the way for future research to invest in the respective examinations.

The effect of narcissism on drive for muscularity was investigated only in Sample 2, and the results were contrary to those from previous (manifest) analyses [5]. Although our analyses were based on larger sample sizes and a more reliable latent analysis approach, future studies should reexamine this question. Here, it is especially necessary to clarify the role that agentic narcissism plays in muscularity-related body image concerns.

Next, both samples mainly consisted of women in their 20s and 30s (*Mdn*_Sample 1_ = 30, *Mdn*_Sample 1_ = 24). This should be kept in mind as levels of narcissism [102, 103] and thinness- and muscularity-related body image concerns [4, 104] decrease with age. Therefore, the relationship between narcissism and body image concerns may also change over the lifespan. Recruiting a sample with greater variability in age and controlling for age effects would hence provide important differentiating insights. Similarly, future research could aim for greater cultural diversity, by expanding the analyses, for example, from individualistic cultures (such as the here examined samples from Spain and Germany) to collectivistic cultures.

Our results did not depend on whether individuals who exceeded EAT13 and BMI cut-offs for ED were included or excluded (see S2 Text). Nonetheless, re-examinations within clinical samples (e.g., individuals with diagnosed eating disorders or muscle dysmorphia) should be realized in future investigations, especially with regard to the outlined implications for clinical practice. Further, our results did not depend on actual body sizes (see S2 Table and S2 Fig). Still, it would be worthwhile for future investigations to consider the degree to which individuals’ ideal images and actual appearances match and to analyse whether (directed) differences in this (mis-)match influence the associations between narcissism facets and body image concerns.

We were interested in the effect of narcissism on body image concerns in women specifically. Expanding analyses to men would nonetheless complement the picture and provide further insights into the relation between facets of narcissism and body image concerns. Literature suggests that men differ from women in the extent of drive for thinness and drive for muscularity [15], importance of appearance [105] and in certain facets of narcissism [45]. It would therefore be intriguing to see whether these gender-related differences also lead to different associations among these variables.

We purposefully focused on body image *concerns*, that is, on cognitive and emotional preoccupation with shape and weight. These concerns do not necessarily imply respective *behaviors* (although they are strong predictors of such; [15, 16, 79]. Thus, future investigations could expand the focus of interest from thinness- and muscularity-related concerns to respective behaviors.

The scope of this paper was a differentiated analysis of the effect of narcissism on body image concerns while considering the importance of appearance as moderator and/or mediator. Since in the past, other personality traits (e.g., neuroticism [40, 41], perfectionism [42]) have been identified as risk factors for body image concerns, too, it would be worthwhile to examine the role importance of appearance in these relations as-well.

Finally, investigating not only drive for thinness but also drive for muscularity importantly accounted for recent developments in female body ideals; however, we did not include another body ideal: the lean ideal, implying defined (but not bulky) muscles and low (but not extremely low) body fat [23, 106, 107]. The corresponding *drive for leanness* is considered distinct from drive for muscularity and drive for thinness and as less harmful than them (e.g., [106]). It would therefore be particularly intriguing to test whether its associations with the facets of narcissism are comparable to drive for thinness and muscularity or instead emerge in a unique pattern.

## Conclusion

The present work examined the relations between the facets of narcissism (i.e., agentic, antagonistic, neurotic) and different types of body image concerns (i.e., drive for thinness, drive for muscularity). On the basis of latent SEM applied to self-report data from two independent nonclinical female samples, we found robust evidence of a strong association between neurotic narcissism and both drive for thinness and drive for muscularity: Women with a stronger tendency toward narcissistic insecurity, hypersensitivity to criticism, and the experience of shame worried more about their body image. Moreover, this link between narcissism and body image concern was mediated (but not consistently moderated) by importance of appearance. These results underline the utility of distinguishing between the narcissism facets when considering predictors of body image concerns and highlight similarities and differences in thinness- versus muscularity-related concerns. They also point out the relevant mechanisms that might be important to tackle in prevention endeavors and clinical practice: Specifically, to reduce body image concerns, interventions might need to be tailored to address the appearance-related cognitive-affective biases that are typical of neurotic narcissists and how they translate into dysfunctional coping mechanisms. Future empirical examinations might aim to apply longitudinal or experimental designs, gather more diverse samples, inspect agentic narcissism in the context of drive for muscularity more thoroughly, expand analyses to actual behaviors, and consider drive for leanness as an additional aspect of body image concerns.

## Supporting information

S1 Table Descriptive statistics for Samples 1 and 2. *Note*. Min = Minimum, Max = Maximum. S1 = Sample 1, S2 = Sample 2. NPI LA = Narcissistic Personality Inventory Leadership/Authority, NPI GE = Grandiose Exhibitionism, NPI EE = Entitlement/ Exploitativeness, NARQ ADM = Narcissistic Admiration and Rivalry Questionnaire Admiration, NARQ RIV = Rivalry, HSNS = Hypersensitive Narcissism Scale, FFNI D = Five Factor Narcissism Inventory Distrust, FFNI NFA = Need for Admiration, FFNI RA = Reactive Anger, FFNI S = Shame, EDI DT = Eating Disorder Inventory Drive for thinness, DMS = Drive for muscularity (cognitive) scale, ASI = Appearance Schema Inventory. 1, 2, and 4 reflect agentic, 6, 8, and 10 neurotic, and 3, 5, 7, 9 antagonistic narcissism.
(DOCX)

S2 Table Control for BMI. (DOCX)

S1 Text Description of the translation process of the NARQ into Spanish. (DOCX)

S2 Text Supplemental analyses of influences of exceeding eating disorder cut-offs. (DOCX)

S1 Fig Illustration of the effect of BMI. Variables were *z*-standardized.
(TIF)

S2 Fig Mediating effect of importance of appearance on the relation between neurotic narcissism and drive for muscularity in Sample 2. Neurotic narcissism, importance of appearance, and drive for muscularity were latent variables (for indicator variables, see Analytical Strategy in the main document). ab = indirect effect, c = direct effect, c’ = total effect. Confidence intervals (CI) were computed using bootstrapping. Path coefficients are unstandardized and printed in bold if significant (*p* < .05).
(TIF)

S3 Fig Simple slopes of high, average, and low importance of appearance moderating the relation between neurotic narcissism and drive for thinness in Samples 1 and 2. The importance of appearance variable was transformed to categorial with Average = average level, High = 1 SD above average level, and Low = 1 SD below average level. Variables of neurotic narcissism and drive for thinness were *z*-standardized.
(TIF)

S4 Fig Simple slopes of high, average, and low importance of appearance moderating the relation between neurotic narcissism and drive for muscularity in Sample 2. The importance of appearance variable was transformed to categorial with Average = average level, High = 1 SD above average level, and Low = 1 SD below average level. Variables of neurotic narcissism and drive for thinness were *z*-standardized.
(TIF)
