# Supplementary material for: Disentangling the contributions of agentic, antagonistic, and neurotic narcissism to drive for thinness and drive for muscularity
Source: PLoS One. 2021 Jun 15;16(6):e0253187. doi: 10.1371/journal.pone.0253187 (PMC8205145; doi:10.1371/journal.pone.0253187)
Supplement: S1 Table — Note. Min = Minimum, Max = Maximum. S1 = Sample 1, S2 = Sample 2. NPI LA = Narcissistic Personality Inventory Leadership/Authority, NPI GE = Grandiose Exhibitionism, NPI EE = Entitlement/ Exploitativeness, NARQ ADM = Narcissistic Admiration and Rivalry Questionnaire Admiration, NARQ RIV = Rivalry, HSNS = Hypersensitive Narcissism Scale, FFNI D = Five Factor Narcissism Inventory Distrust, FFNI NFA = Need for Admiration, FFNI RA = Reactive Anger, FFNI S = Shame, EDI DT = Eating Disorder Inventory Drive for thinness, DMS = Drive for muscularity (cognitive) scale, ASI = Appearance Schema Inventory. 1, 2, and 4 reflect agentic, 6, 8, and 10 neurotic, and 3, 5, 7, 9 antagonistic narcissism. (DOCX) [file pone.0253187.s001.docx]

**S1 Table. Descriptive Statistics for Samples 1 and 2.**

|  | Variable | *M* | | *SD* | | Min | | Max | | Cronbach’s α | |
| --- | --- | --- | --- | --- | --- | --- | --- | --- | --- | --- | --- |
|  |  | S1 | S2 | S1 | S2 | S1 | S2 | S1 | S2 | S1 | S2 |
| 1 | NPI LA | 0.45 | 0.32 | 0.21 | 0.21 | 0.00 | 0.00 | 1.00 | 0.91 | .61 | .68 |
| 2 | NPI GE | 0.31 | 0.30 | 0.22 | 0.22 | 0.00 | 0.00 | 1.00 | 0.90 | .66 | .70 |
| 3 | NPI EE | 0.28 | 0.25 | 0.26 | 0.25 | 0.00 | 0.00 | 1.00 | 1.00 | .35 | .37 |
| 4 | NARQ ADM | 3.23 | 2.86 | 0.90 | 0.83 | 1.33 | 1.00 | 5.78 | 5.11 | .83 | .85 |
| 5 | NARQ RIV | 1.95 | 2.01 | 0.72 | 0.71 | 1.00 | 1.00 | 4.89 | 5.22 | .78 | .80 |
| 6 | HSNS | 2.80 | 2.96 | 0.60 | 0.49 | 1.20 | 1.70 | 4.70 | 4.10 | .74 | .59 |
| 7 | FFNI D | - | 2.62 | - | 0.78 | - | 1.00 | - | 4.75 | - | .65 |
| 8 | FFNI NFA | - | 3.00 | - | 0.91 | - | 1.00 | - | 5.00 | - | .74 |
| 9 | FFNI RA | - | 2.77 | - | 0.85 | - | 1.00 | - | 4.75 | - | .73 |
| 10 | FFNI S | - | 3.46 | - | 0.91 | - | 1.00 | - | 5.00 | - | .80 |
| 11 | EDI DT | 3.23 | 2.88 | 1.43 | 1.39 | 1.00 | 1.00 | 6.00 | 6.00 | .91 | .94 |
| 12 | DMS | - | 2.23 | - | 0.96 | - | 1.00 | - | 5.43 | - | .87 |
| 13 | ASI | 3.15 | 3.21 | 0.74 | 0.64 | 2.22 | 1.06 | 5.00 | 4.95 | .93 | .90 |

*Note.* Min = Minimum, Max = Maximum. S1 = Sample 1, S2 = Sample 2. NPI LA = Narcissistic Personality Inventory Leadership/Authority, NPI GE = Grandiose Exhibitionism, NPI EE = Entitlement/ Exploitativeness, NARQ ADM = Narcissistic Admiration and Rivalry Questionnaire Admiration, NARQ RIV = Rivalry, HSNS = Hypersensitive Narcissism Scale, FFNI D = Five Factor Narcissism Inventory Distrust, FFNI NFA = Need for Admiration, FFNI RA = Reactive Anger, FFNI S = Shame, EDI DT = Eating Disorder Inventory Drive for thinness, DMS = Drive for muscularity (cognitive) scale, ASI = Appearance Schema Inventory. 1, 2, and 4 reflect agentic, 6, 8, and 10 neurotic, and 3, 5, 7, 9 antagonistic narcissism.
