# Supplementary material for: Disentangling the contributions of agentic, antagonistic, and neurotic narcissism to drive for thinness and drive for muscularity
Source: PLoS One. 2021 Jun 15;16(6):e0253187. doi: 10.1371/journal.pone.0253187 (PMC8205145; doi:10.1371/journal.pone.0253187)
Supplement: S2 Table — (DOCX) [file pone.0253187.s002.docx]

**S2 Table. Control for BMI.**

|  | Sample 1 | |  | Sample 2 | | | |
| --- | --- | --- | --- | --- | --- | --- | --- |
|  | Drive for thinness | |  | Drive for thinness | | Drive for muscularity | |
|  | β | *p* |  | β | *p* | β | *p* |
| Neurotic narcissism | **.43** | <.001 |  | **.47** | <.001 | **.26** | <.001 |
| BMI | **.28** | <.001 |  | **.14** | .003 | .04 | .446 |
| Neurotic narcissism   x BMI | -.05 | .382 |  | -.00 | .985 | -.03 | .660 |
| *R*² (adjusted) | 27% (26%) | |  | 24% (23%) | | 7% (6%) | |
| *Note.* β = Standardized regression coefficients. Significant coefficients (*p* < .05) in bold. | | | | | | | |
