# Supplementary material for: Disentangling the contributions of agentic, antagonistic, and neurotic narcissism to drive for thinness and drive for muscularity
Source: PLoS One. 2021 Jun 15;16(6):e0253187. doi: 10.1371/journal.pone.0253187 (PMC8205145; doi:10.1371/journal.pone.0253187)
Supplement: S1 Text — (DOCX) [file pone.0253187.s003.docx]

**S1 Text. Description of The Translation Process of the NARQ Into Spanish.**

The translation process was realized as forward/backward translation process that consisted of five consecutive steps (Forward Translation, Revision, Backward Translation, Revision, Approval). First, a bilingual (European Spanish/German) native speaker translated the German version of the NARQ into an initial Spanish version. Second, a bilingual (European Spanish/German) psychologist with expertise in questionnaire construction and psychometrics revised this initial forward-translation to optimize the match between the Spanish and original German items. Third, another bilingual (European Spanish/German) native speaker translated the revised Spanish version of the NARQ back into a German version.Fourth, another bilingual (European Spanish/German) psychologist with expertise in questionnaire construction and psychometrics revised this initial back-translation to optimize the match between the Spanish and translated German items. Fifth, the bilingual (European Spanish/German) psychologist and the third author of this study compared the German back-translation against the German original and approved this version.
