# Supplementary material for: Disentangling the contributions of agentic, antagonistic, and neurotic narcissism to drive for thinness and drive for muscularity
Source: PLoS One. 2021 Jun 15;16(6):e0253187. doi: 10.1371/journal.pone.0253187 (PMC8205145; doi:10.1371/journal.pone.0253187)
Supplement: S2 Text — (DOCX) [file pone.0253187.s004.docx]

**S2 Text. Supplemental Analyses of Influences of Exceeding Eating Disorder Cut-Offs.**

We tested whether there would be any differences between participants who reported cut-off exceeding eating disorder symptoms (i.e., BMI < 17 kg/m² and EAT13 sum score > 10) and participants who did not exceed these criteria of clinical relevance. In Sample 1, nobody matched the BMI criterion, so we could test for differences only in Sample 2. In Sample 2, six participants reported both, a BMI lower than 17 and an EAT13 sum score higher than 10. We removed these six participants from the data set and reconducted the main SEM analyses. The results were similar to the main findings for drive for thinness (neurotic narcissism: β = .56, *p* <.001, agentic narcissism: β = .07, *p* =.652, antagonistic narcissism: β = .01 p=.951). This indicates that the main results were not particularly driven by individuals who matched ED criteria.
