# Supplementary figures and images for: Disentangling the contributions of agentic, antagonistic, and neurotic narcissism to drive for thinness and drive for muscularity
Source: PLoS One. 2021 Jun 15;16(6):e0253187. doi: 10.1371/journal.pone.0253187 (PMC8205145; doi:10.1371/journal.pone.0253187)

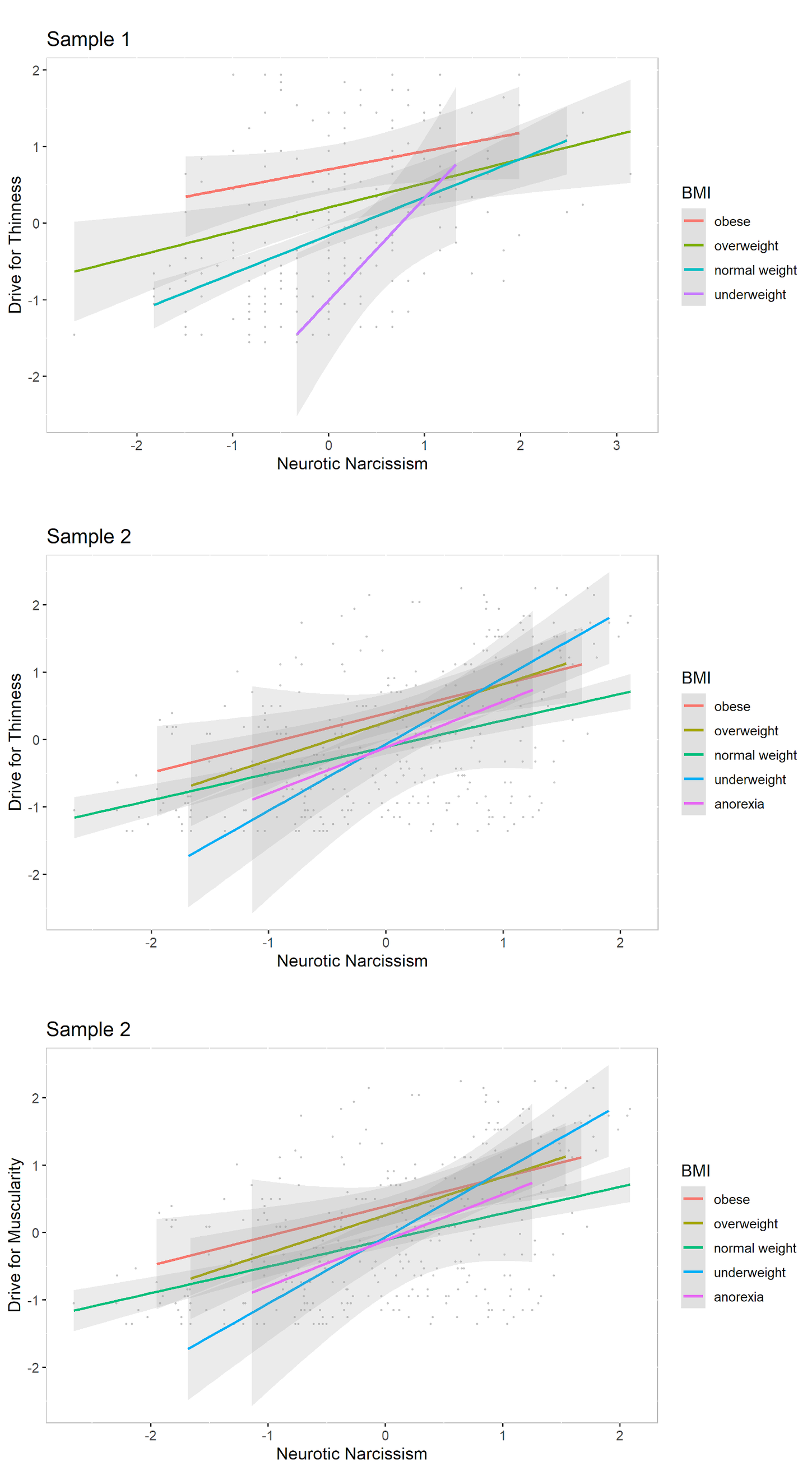

Supplement: S1 Fig — Variables were z-standardized. (TIF) [file pone.0253187.s005.tif]

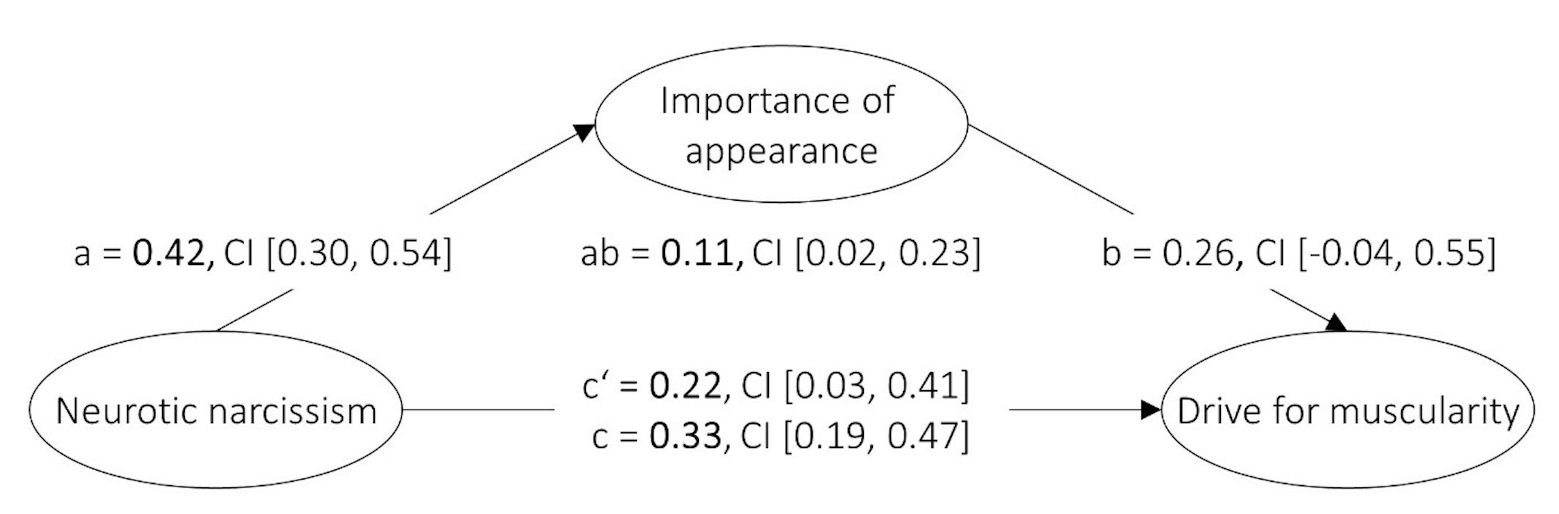

Supplement: S2 Fig — Neurotic narcissism, importance of appearance, and drive for muscularity were latent variables (for indicator variables, see Analytical Strategy in the main document). ab = indirect effect, c = direct effect, c’ = total effect. Confidence intervals (CI) were computed using bootstrapping. Path coefficients are unstandardized and printed in bold if significant (p < .05). (TIF) [file pone.0253187.s006.tif]

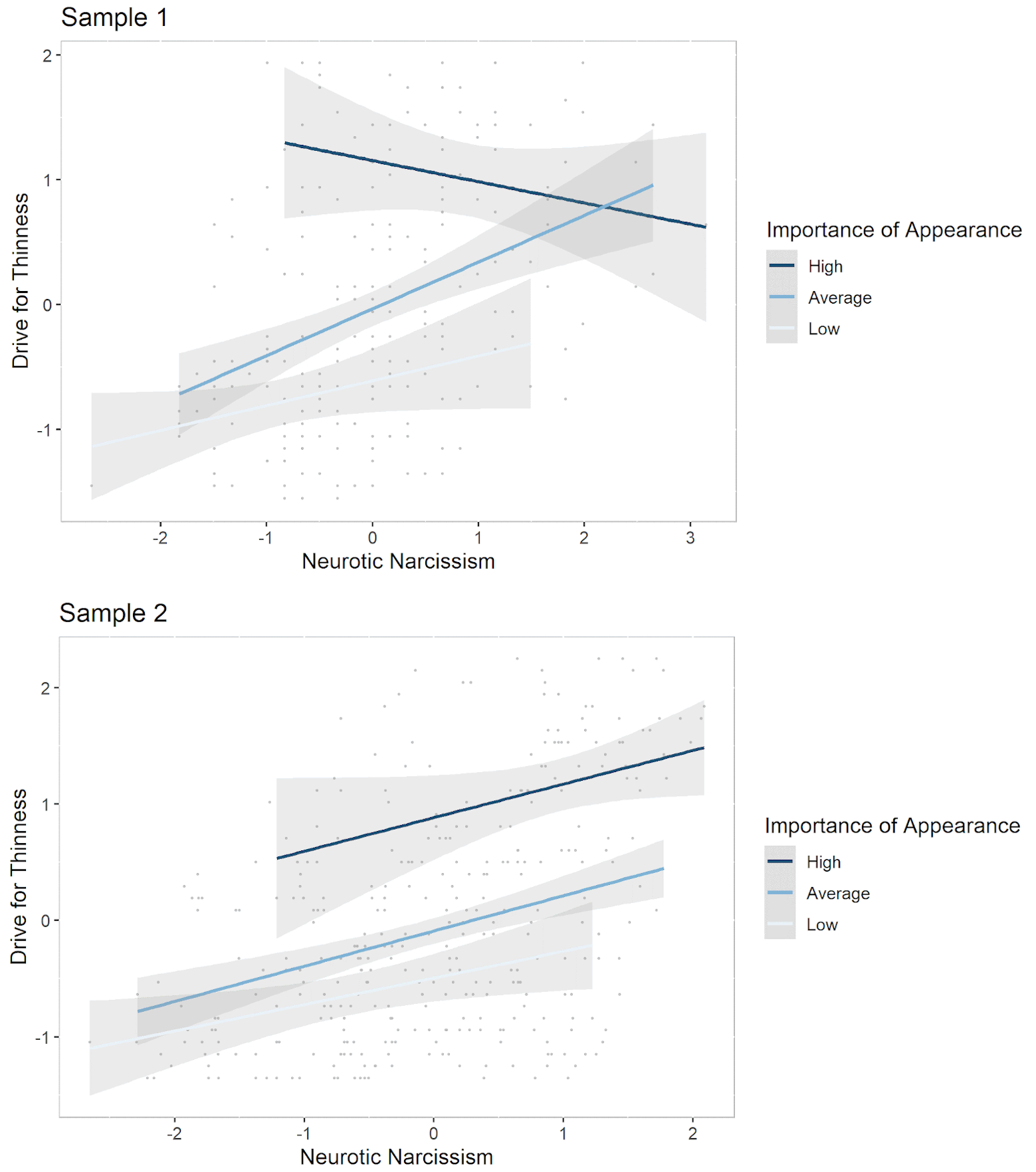

Supplement: S3 Fig — The importance of appearance variable was transformed to categorial with Average = average level, High = 1 SD above average level, and Low = 1 SD below average level. Variables of neurotic narcissism and drive for thinness were z-standardized. (TIF) [file pone.0253187.s007.tif]

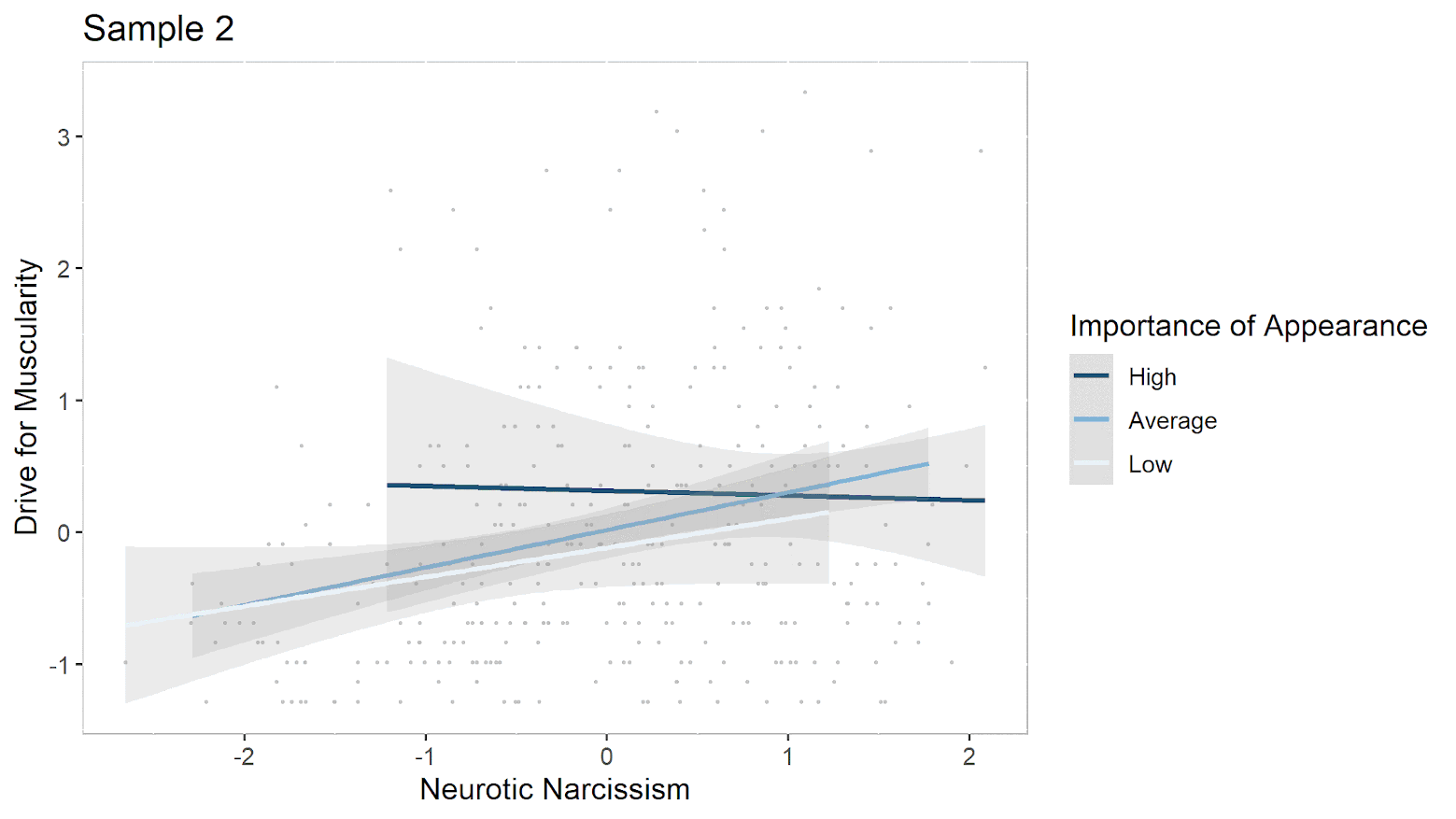

Supplement: S4 Fig — The importance of appearance variable was transformed to categorial with Average = average level, High = 1 SD above average level, and Low = 1 SD below average level. Variables of neurotic narcissism and drive for thinness were z-standardized. (TIF) [file pone.0253187.s008.tif]
